# Menopausal Impact on the Association Between Thyroid Dysfunction and Lipid Profiles: A Cross-Sectional Study

**DOI:** 10.3389/fendo.2022.853889

**Published:** 2022-03-14

**Authors:** Yutong Han, Chuyuan Wang, Lihui Zhang, Jun Zhu, Mei Zhu, Yongze Li, Di Teng, Weiping Teng, Zhongyan Shan

**Affiliations:** ^1^ Department of Endocrinology and Metabolism, The Institute of Endocrinology, National Health Commission (NHC) Key Laboratory of Diagnosis and Treatment of Thyroid Diseases, The First Hospital of China Medical University, Shenyang, China; ^2^ Department of Endocrinology, Second Hospital of Hebei Medical University, Shijiazhuang, China; ^3^ Department of Endocrinology, The First Affiliated Hospital of Xinjiang Medical University, Urumqi, China; ^4^ Department of Endocrinology and Metabolism, Tianjin Medical University General Hospital, Tianjin, China

**Keywords:** thyroid function, subclinical hypothyroidism, blood lipids, dyslipidemia, hypercholesterolemia, menopause

## Abstract

**Background:**

Both dyslipidemia and thyroid dysfunction have a high prevalence rate and are important risk factors for cardiovascular diseases. However, the relationship between blood lipids and thyroid dysfunction is still controversial. This study aims to analyze the blood lipids in people with different thyroid functions.

**Methods:**

A total of 80937 adults were included in this population-based cross-sectional TIDE survey, which collected demographic and clinical data on thyroid function, blood lipid levels and other metabolic indicators. After screening, the final analysis included 10,747 participants, who were divided into hypothyroidism (n=311), subclinical hypothyroidism (n=5015), hyperthyroidism (n=203), subclinical hyperthyroidism (n=118) and control (n=5100) groups. The risk of dyslipidemia was analyzed by a logistic regression model and divided into groups of female menopausal.

**Results:**

After full adjustment, significant associations were found between hypothyroidism and hypertriglyceridemia. Subclinical hypothyroidism was associated with a significantly higher risk of hypertriglyceridemia and hyper-low density lipoprotein cholesterolemia. Hyperthyroidism was significantly correlated with a reduced risk of hypercholesterolemia and hyper-low density lipoprotein cholesterolemia but positively correlated with the risk of low-high density lipoprotein cholesterolemia. There was no significant association between subclinical hyperthyroidism and blood lipids. Hypothyroidism increased the risk of hypertriglyceridemia in both premenopausal and postmenopausal females. Subclinical hypothyroidism was significantly associated with increased hypertriglyceridemia and low-high density lipoprotein cholesterolemia in premenopausal females. Hyperthyroidism was significantly associated with a reduced risk of hypercholesterolemia and hyper-low density lipoprotein cholesterolemia in premenopausal females and an increased risk of low-high density lipoprotein cholesterolemia in postmenopausal female.

**Conclusion:**

Abnormal thyroid function has an important effect on blood lipids and is closely related to female menopause.

## 1 Introduction

Thyroid dysfunction is the most common disease in humans; the prevalence in China is as high as 15.22% and shows an upward trend (1). Thyroid hormones maintain the normal metabolism of blood lipids by participating in synthesis, transport, degradation and other processes (2). Dyslipidemia is an important risk factor for arteriosclerotic cardiovascular disease (ASCVD). Although current research is still controversial, most literature has reported that the levels of total cholesterol (TC), triglyceride (TG) and low-density lipoprotein cholesterol (LDL-C) are increased in clinical hypothyroidism and decreased in clinical hyperthyroidism (3, 4). Changes in the lipid profile in subclinical thyroid function have also been extensively reported, but there is no consistent conclusion. In some studies, no significant changes in lipids were observed in patients with subclinical hypothyroidism compared with patients with normal thyroid function (5). However, Duntas et al. revealed that subclinical hypothyroidism patients with thyroid stimulating hormone (TSH)> 10 mU/L had higher levels of TC and LDL-C and lower levels of high-density lipoprotein cholesterol (HDL-C) (6). The Norwegian HUNT study proved that even in people with normal thyroid function, TC, LDL-C, and TG increased with increasing TSH, while HDL-C decreased with increasing TSH (7).

In addition to thyroid dysfunction, other important risk factors, such as age, sex and menopause, also affect the relationship between thyroid function and blood lipids. Through stepwise regression analysis, Tognini et al. proved that age was the most important factor affecting TC, and TSH was the second most important factor after age (8). Delitala et al. found that the relationship between TSH levels and blood lipids is affected by estrogen levels (9). Although many studies have been conducted on the relationship between thyroid dysfunction and dyslipidemia, further in-depth analysis is still needed due to various confounding factors and inconsistent results. Based on the data of the Thyroid disorders, Iodine status and Diabetes Epidemiological (TIDE) survey in China, the blood lipid profiles of different thyroid functional states were analyzed, in order to comprehensively analyze the effect of thyroid function on blood lipids and its risk factors.

## 2 Materials and Methods

### 2.1 Study Population

**Figure 1:**
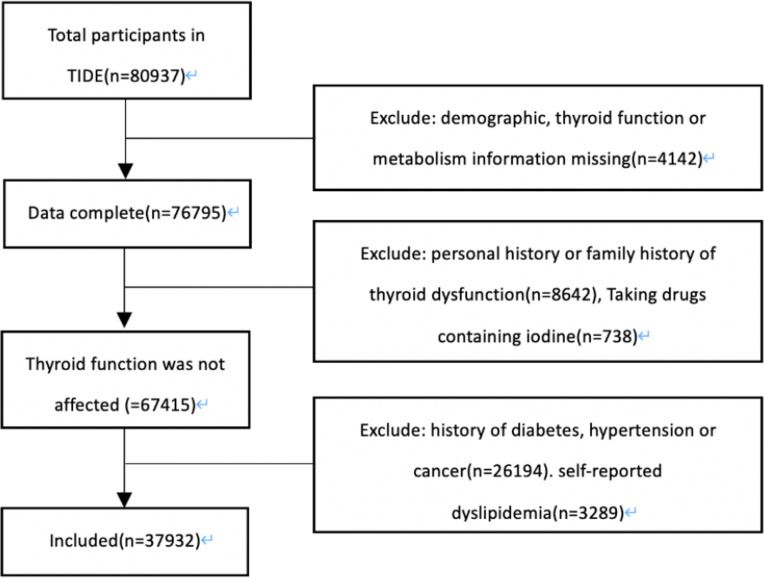
Screening flow chart. TIDE, Thyroid Disorders, Iodine Status and Diabetes, a national epidemiological cross- sectional study.

The data were based on the TIDE project, which is a national cross-sectional survey covering 31 provinces in China (1). The inclusion criteria of the subjects were as follows: age 18 years and above; local residence for at least five years; no iodine-containing drugs or contrast media within three months; and nonpregnant women. Our research program was approved by the Ethics Committee of China Medical University. All the respondents signed the informed consent form. Finally, a total of 80,937 cases were investigated. As shown in **Figure 1**, excluding patients with incomplete data, a family and personal history of thyroid disorder, the taking of drugs affecting thyroid function, diabetes, hypertension and self-reported dyslipidemia. There were 311 cases of hypothyroidism, 5015 cases of subclinical hypothyroidism, 118 cases of subclinical hyperthyroidism and 203 cases of hyperthyroidism. A total of 5100 patients with normal thyroid function were randomly selected as the control group.

### 2.2 Research Methods

All subjects completed questionnaires including demographic information, personal and family history of thyroid disease, personal history of diabetes, hypertension, cancer, and dyslipidemia. Weight, height and blood pressure were measured by trained investigators according to the standard scheme. Body mass index (BMI) was calculated by dividing weight (kg) by height (m) squared (kg/m2). Using an OMRON electronic blood pressure monitor (Omron HEM-7430, Omron Corporation), the blood pressure was measured twice in a row on the nondominant arm, and the mean was calculated and used for analysis.

Fasting venous blood samples were collected at least 8 hours after fasting. All the specimens were airlifted to the central laboratory to complete unified testing. Serum TSH, thyroid peroxidase antibodies (TPOAb) and thyroglobulin antibodies (TgAb) were measured in all samples. FT4 and free triiodothyronine (FT3) were measured in the samples with TSH < 0.27 mIU/L, and FT4 was measured in the samples with TSH > 4.2 mIU/L. Serum TSH, FT3, FT4, TPOAb and TgAb were determined by electrochemiluminescence immunoassay (Cobas 601 analyzer, Roche Diagnostic, Switzerland). TG, TC, LDL-C and HDL-C were determined by an automatic biochemical analyzer (Mindray BS-180 analyzer). Fasting plasma glucose (FPG) and the oral glucose tolerance test (OGTT)-2 hPG were determined by the hexokinase method (Au400 automatic analyzer, Olympus, Japan).

### 2.3 Diagnostic Criteria

#### 2.3.1 Abnormal Thyroid Function

Overt hyperthyroidism: TSH < 0.27 mIU/L; FT4 > 22 pmol/L or FT3 > 6.8 pmol/L.

Subclinical hyperthyroidism: TSH < 0.27 mIU/L; FT4 and FT3 within the normal range.

Overt hypothyroidism: TSH > 4.2 mIU/L; FT4 < 12 pmol/L.

Subclinical hypothyroidism: TSH > 4.2 mIU/L; FT4 within the normal range.

#### 2.3.2 Dyslipidemia

According to the diagnostic criteria of the National Cholesterol Education Program Adult Treatment Panel III (10):

Hypertriglyceridemia (HyperTG): TG ≥ 1.7 mmol/L.

Hypercholesterolemia (HyperTC): TC ≥ 5.2 mmol/L.

Hyper LDL-C: LDL-C ≥ 3.4 mmol/L.

Low HDL-C: Male: HDL-C < 1.0 mmol/L.

Female: HDL-C < 1.3 mmol/L.

#### 2.3.3 Diabetes Mellitus

According to the 2018American Diabetes Association(ADA) diabetes diagnosis and treatment criteria (11), the presence of one of the following conditions is diagnosed as diabetes: 1) FPG ≥ 7.0 mmol/L 2) OGTT-2h PG ≥ 11.1 mmol 3) HbA1c ≥ 6.5%.

#### 2.3.4 Hypertension

According to the 2018 European Society of Cardiology and the European Society of Hypertension (ESC/ESH) guidelines for the management of hypertension (12), hypertension is diagnosed by any one of the following 2 items: systolic blood pressure (SBP) ≥ 140 mmHg or diastolic blood pressure (DBP) ≥ 90 mmHg.

#### 2.3.5 Statistical Method

Continuous variables with a normal distribution are described by the mean (standard deviation), Student’s t test was used for comparisons between the two groups, nonnormally distributed variables were described by the median (quartile spacing), and the Mann-Whitney U test was used for comparisons between the two groups. Categorical data are presented as counts and percentages, and the chi-square test was used for comparisons between groups. The odds ratio (OR) value and 95% confidence interval (CI) were calculated by multivariate logistic regression to evaluate the relationship between thyroid disease and blood lipids. P < 0.05 was considered statistically significant. All statistics were calculated by SPSS, version 23.0 (IBM, USA).

## 3 Result

### 3.1 Basic Characteristics of the Study Population

As shown in **Table 1**, a total of 10,751 subjects were studied there were significant differences in age, sex distribution, nationality, smoking, education, BMI, SBP, DBP, FBG, OGTT-2hPG and HbA1c among the groups (all p<0.05). In terms of thyroid function, the level of TSH was significantly higher in the hypothyroidism group and subclinical hypothyroidism group (p=0.000) but significantly lower in the hyperthyroidism group and subclinical hyperthyroidism group (p=0.000). The levels of TgAb and TPOAb in each disease group were significantly higher than control group (p < 0.05). In terms of blood lipids, the level of TC was the highest in the hypothyroidism group and the lowest in the hyperthyroidism group (p<0.05). The hypothyroidism group and subclinical hypothyroidism group presented with significantly higher serum levels of TG than the control group (p<0.05). The level of LDL-C in the hyperthyroidism group was significantly lower than control group (p<0.05). Our study also revealed a significant reduction in HDL-C only in the subclinical hypothyroidism group and hyperthyroidism group (p < 0.01).

**Table 1:** Baseline characteristics of study participants.

	Ohypo (n=311)	SHypo (n=5015)	Euthyroid (n=5100)	SHyper (n=118)	OHyper (n=203)
Age (years)	45.37 ± 14.61^*^	39.72 ± 14.58^*^	37.32 ± 13.21	42.36 ± 15.20^*^	39.23 ± 13.44
Gender					
Male[n ( %)]	69 (22.2)*	1868 (37.2)*	1759 (34.5)	37 (31.4)	75 (36.9)
Female[n ( %)]	242 (77.8)*	3147 (62.8)*	3341 (65.5)	81 (68.5)	128 (63.1)
Smoke[n ( %)]	40 (12.9)*	882 (17.6)	988 (19.4)	25 (21.2)	46 (22.7)
Ethnicity					
Han[n ( %)]	248 (79.7)*	4378 (87.3)*	4569 (89.6)	99 (83.9)	184 (90.6)
Others[n ( %)]	63 (20.3)*	637 (12.7)*	531 (10.4)	19 (16.1)	19 (9.4)
Education					
Less than university[n ( %)]	248 (80.0)*	3342 (66.7)*	3097 (60.9)	84 (71.2)	139 (68.5)
university[n ( %)]	62 (20.0)*	1667 (33.3)*	1993 (39.1)	34 (28.8)	64 (31.5)
BMI (kg/m2)	23.79 ± 3.17^*^	23.16 ± 3.56^*^	22.83 ± 3.46	23.15 ± 3.40	22.81 ± 4.04
SBP (mmHg)	119 (110-128)*	118 (110-125)*	116 (107-124)	120 (111-129)*	119 (110-127)
DBP (mmHg)	74 (69-80)*	74 (69-80)*	72 (67-79)	75 (68-80)	72 (67-80)
FBG (mmol/l)	5.00 (4.66-5.40)	4.95 (4.59-5.31)	4.96 (4.60-5.30)	4.98 (4.71-5.30)	5.11 (4.64-5.55)*
2hPG (mmol/l)	5.48 (4.54-6.70)	5.50 (4.67-6.43)	5.57 (4.71-6.50)	5.46 (4.87-6.51)	5.95 (4.91-7.23)*
HbA1c (%)	5.40 (5.10-5.70)*	5.30 (5.10-5.60)*	5.30 (5.00-5.60)	5.30 (5.10-5.60)	5.30 (5.10-5.70)
TC (mmol/l)	4.55 (3.89-5.36)^*^	4.38 (3.80-5.05)	4.39 (3.80-5.05)	4.30 (3.54-5.05)	3.93 (3.30-4.45)^*^
TG (mmol/l)	1.20 (0.84-1.74)*	1.12 (0.80-1.62)^*^	0.99 (0.72-1.47)	1.08 (0.74-1.57)	1.04 (0.79-1.39)
LDL-C (mmol/l)	2.60 (2.05-3.22)	2.55 (2.08-3.08)	2.50 (2.05-3.05)	2.37 (1.97-2.99)	2.21 (1.74-2.68)*
HDL-C (mmol/l)	1.45 (1.21-1.70)	1.42 (1.18-1.71)*	1.46 (1.22-1.74)	1.48 (1.21-1.90)	1.37 (1.17-1.65)*
TSH (mIU/l)	8.39 (5.53-18.27)*	5.28 (4.64-6.49)*	2.15 (1.55-2.88)	0.11 (0.04-0.19)*	0.01 (0.01-0.06)*
TgAb (IU/mL)	127.90 (15.85-441.60)*	15.49 (11.84-29.26)*	14.77 (11.39-19.53)	21.35 (15.06-223.25)*	74.97 (16.63-397.00)*
TPOAb (IU/mL)	33.06 (10.41-370.50)*	11.18 (7.83-17.51)*	10.09 (7.17-14.40)	12.75 (8.95-70.94)*	20.92 (10.30-171.70)*

*Significantly different from control (p<0.05).

OHypo, overt-hypothyroidism; SHypo, subclinical-hypothyroidism; SHyper, subclinical-hyperthyroidism; OHyper, overt-hyperthyroidism; BMI, body mass index; SBP, systolic blood pressure; DBP, diastolic blood pressure; FBG, fasting blood glucose; OGTT, oral glucose tolerance test; HbA1c, glycosylated hemoglobin; TC, total cholesterol; TG, triglyceride; LDL-C, low-density lipoprotein cholesterol; HDL-C, high-density lipoprotein cholesterol; TSH, thyroid stimulating hormone; TgAb, thyroglobulin antibody; TPOAb, thyroid peroxidase antibody.

### 3.2 Comparison of the Prevalence of Dyslipidemia in Different Thyroid Function Statuses

**Figure 2:**
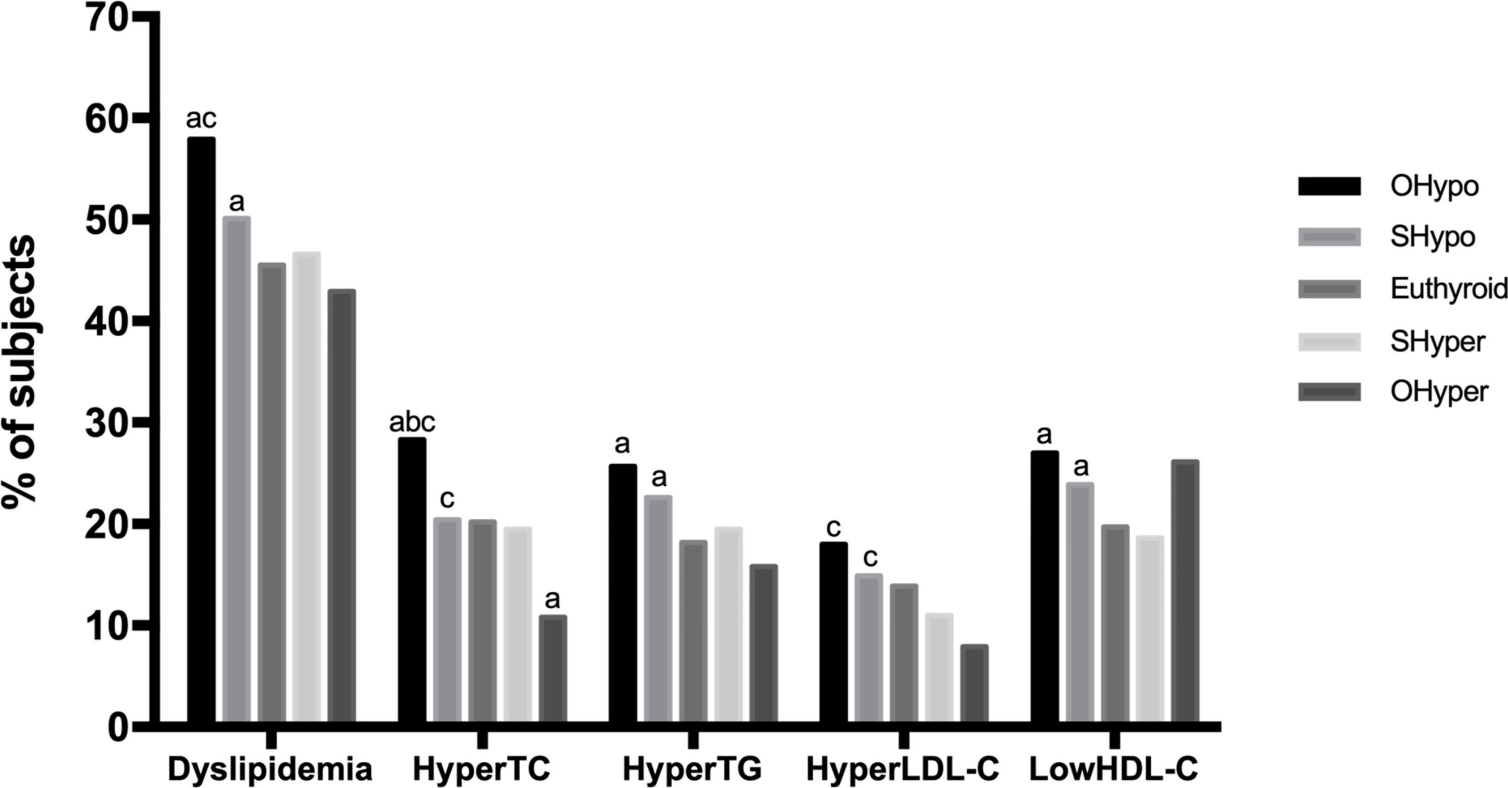
Comparison of the prevalence of dyslipidemia based on thyroid function. HyperTC, hypercholesterolemia; HyperTG, hypertriglyceridemia; HyperLDL-C, high serum level of low-density; LowHDL-C, low serum level of high-density lipoprotein-cholesterol. a, Compared with control, the prevalence was significantly different (p<0.005) b, Compared with SHypo, the prevalence was significantly different (p<0.005) c, Compared with OHyper, the prevalence was significantly different (p<0.005).

As shown in **Figure 2**, the prevalence of dyslipidemia in the hypothyroidism and subclinical hypothyroidism groups was significantly higher than control group (p=0.000; p=0.000). Compared with control group, the prevalence of hyperTC in the hypothyroidism group was significantly higher (p=0.000), but in the hyperthyroidism group, it was significantly lower (p =0.001). The hypothyroidism and subclinical hypothyroidism groups presented with a significantly higher prevalence of hyperTG. Moreover, no significant difference was observed in the prevalence of hyperLDL-C between the thyroid disorder group and the control group. Our study also showed that the prevalence of low HDL-C in the hypothyroidism and subclinical hypothyroidism groups was significantly higher than that in the control group (p < 0.05).

In the disease spectrum of hypothyroidism-subclinical hypothyroidism-normal control-subclinical hyperthyroidism-hyperthyroidism, the prevalence rates of dyslipidemia, hyperTG, hyperLDL-C and hyperthyroidism decreased linearly (p = 0.000), and the prevalence of hypoHDL-C showed a U-shaped curve (p = 0.000).

### 3.3 Analysis of Risk Factors for Dyslipidemia

As shown in **Table 2**, logistic regression was used to analyze the risk factors for dyslipidemia. In univariate analysis, hypothyroidism was positively correlated with dyslipidemia and all subgroups of dyslipidemia. After adjusting for age, sex, nationality, smoking, education, BMI, SBP, DBP, FBG, OGTT-2hPG, HbA1c, TPOAb and TgAb, significant associations were found only between hypothyroidism and hyperTG (OR=1.502 95% CI: 1.124-2.007 p= 0.006). After full adjustment, subclinical hypothyroidism was associated with a significantly higher risk of dyslipidemia (OR=1.115 95% CI: 1.025-1.213 p= 0.011) and was positively correlated with the risk of hyperTG (OR=1.248 95% CI: 1.124-1.368 p=0.000) and hyperLDL-C (OR=1.287, 95% CI: 1.165-1.423, p= 0.000). Hyperthyroidism was significantly correlated with a reduced risk of hyperTC (OR=0.384 95% CI: 0.242-0.610 p=0.000) and hyperLDL-C (OR= 0.412 95% CI: 0.242-0.703 p= 0.001) but positively correlated with the risk of low HDL-C (OR=1.497 95% CI: 1.065-2.104 p=0.020). There was no significant association between subclinical hyperthyroidism and serum lipids.

**Table 2:** Risk factor analysis for dyslipidemia.

	Model	Ohypo	Shypo	Shyper	Ohyper
		OR (95%CI)	OR (95%CI)	OR (95%CI)	OR (95%CI)
Dyslipidemia	1	1.645 (1.305-2.074)*	1.205 (1.114-1.302)*	1.045 (0.725-1.507)	0.898 (0.676-1.192)
	2	1.168 (0.908-1.502)	1.115 (1.025-1.213)*	0.875 (0.582-1.263)	0.770 (0.568-1.043)
HyperTC	1	1.559 (1.207-2.014)*	1.015 (0.921-1.118)	0.957 (0.604-1.516)	0.480 (0.307-0.752)*
	2	1.109 (0.837-1.468)	0.913 (0.823-1.012)	0.758 (0.469-1.224)	0.384 (0.242-0.610)*
HyperTG	1	1.559 (1.197-2.030)*	1.311 (1.189-1.445)*	1.090 (0.687-1.728)	0.842 (0.574-1.237)
	2	1.502 (1.124-2.007)*	1.248 (1.124-1.368)*	1.002 (0.612-1.640)	0.740 (0.490-1.118)
HyperLDL-C	1	1.362 (1.009-1.839)*	1.089 (0.973-1.215)	0.768 (0.429-1.374)	0.531 (0.317-0.890)*
	2	0.964 (0.695-1.338)	0.979 (0.870-1.102)	0.622 (0.342-1.131)	0.412 (0.242-0.703)*
LowHDL-C	1	1.506 (1.162-1.952)*	1.277 (1.162-1.404)*	0.933 (0.584-1.490)	1.438 (1.043-1.982)*
	2	1.253 (0.947-1.659)	1.287 (1.165-1.423)*	0.892 (0.549-1.448)	1.497 (1.065-2.104)*

*P < 0.05.

Model 1: Single-factor analysis.

Model 2: Adjusted for age, sex, ethincity, education level, smoke, BMI, SBP, DBP, FBG, HbA1c ,OGTT-2hPG, TPOAb and TgAb.

### 3.4 Effect of Female Menopause on the Relationship Between Thyroid Function and Blood Lipids

**Figure 3:**
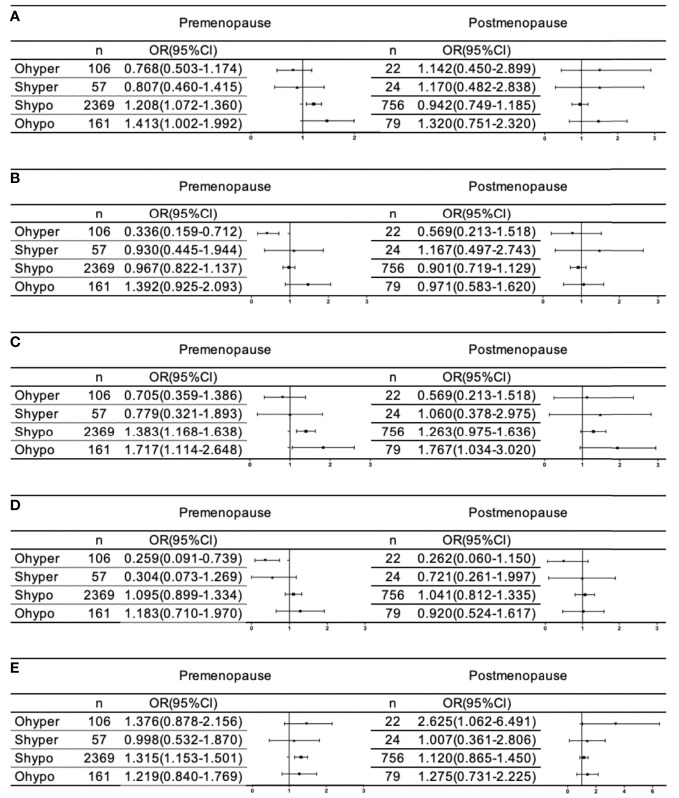
Association of thyroid disorder with dyslipidemia **(A)**, HyperTC **(B)**, HyperTG **(C)**, HyperLDL-C **(D)**, and LowHDL-C **(E)** based on menopause in female. All analyses were adjusted for age, sex, ethincity, education level, smoke, BMI, SBP, DBP, FBG, HbA1C, 2hPG, TPOAb and TgAb.

We further analyzed whether female menopause interacts with the relationship between different thyroid function statuses and blood lipids. As shown in **Figure 3**, after full adjustment, hypothyroidism (OR=1.413 95% CI: 1.002-1.992, p= 0.049) and subclinical hypothyroidism (OR=1.208 95% CI: 1.072-1.360, p=0.002) were significantly associated with an increased risk of dyslipidemia only in premenopausal females, but no significant correlation was found after menopause. Females with hypothyroidism were at significantly higher risk of hyperTG without being affected by menopause. However, subclinical hypothyroidism was associated with a significantly higher risk of hyperTG (OR=1.383 95% CI: 1.168-1.638, p=0.000) and low HDL (OR 1.315 95% CI: 1.153-1.503, p=0.000) in premenopausal females, but no significant association was found after menopause. Hyperthyroidism was significantly associated with a reduced risk of hyperTC (OR=0.336 95% CI: 0.159-0.712 p=0.004) and hyperLDL-C (OR=0.259 95% CI: 0.091-0.739 p=0.012) only in premenopausal female. No correlation was found between hyperthyroidism and low HDL-C before menopause, but there was a significant correlation between hyperthyroidism and the risk of low HDL-C after menopause (OR=2.625 95% CI: 1.062-6.491 p=0.037).

## 4 Discussion

Both thyroid disorder and dyslipidemia have a high prevalence rate and play a particularly critical role in the development of cardiovascular disease, but the relationship between thyroid function and blood lipids is still controversial. Based on the TIDE data, this study found that in the disease spectrum of hypothyroidism-subclinical hypothyroidism-normal control-subclinical hyperthyroidism-hyperthyroidism, the prevalence rates of dyslipidemia, hyperTG, hyperTC and hyperLDL-C showed a decreasing linear trend, while the prevalence of lowHDL-C showed a U-shaped curve. Premenopausal females with hypothyroidism and subclinical hypothyroidism had an increased risk of dyslipidemia, while premenopausal females with hyperthyroidism had a lower risk of hyperTC and hyperLDL-C. In postmenopausal women, a significant association was found between hyperthyroidism and lowHDL-C. There was no significant association between subclinical hyperthyroidism and blood lipids.

Most of the effects of hyperthyroidism on blood lipids are beneficial. In our study, the level of TC in the hyperthyroidism group was significantly lower than that in the other groups, and the prevalence rate of hyperTC and the level of LDL-C in the hyperthyroidism group were significantly decreased. After controlling for confounding factors, the risk of hyperTC and hyperLDL-C in patients with hyperthyroidism decreased by 57.4% and 56.4%, respectively. A mild decrease in TC can reduce the incidence of coronary heart disease by 30% (13). The beneficial effect of hyperthyroidism on blood lipids cannot be ignored. However, the risk of low HDL in patients with hyperthyroidism increased by 47.7%. In line with our results, Kung et al. also found that the levels of TC, LDL-C, and HDL-C in patients with hyperthyroidism were significantly lower than control group (14); Hamlaoui and other studies also reached a similar conclusion (3, 15). Our study analyzed the specific extent of the effect of hyperthyroidism on blood lipids by the regression model, and the results excluded the effect of other confounding factors, which are more convincing. The decrease in HDL-C levels is closely related to the occurrence and development of ASCVD, indicating that the effect of hyperthyroidism on blood lipids is not entirely protective and may be a reason for the adverse effects of hyperthyroidism on the cardiovascular system.

In our study, the effect of subclinical hyperthyroidism on blood lipids was not statistically significant, Razvi S has reported similar conclusions (16). However, it has also been reported that the levels of TC, LDL-C, TG in patients with subclinical hyperthyroidism are higher (17), the difference mainly due to the older age of the study population. Considering the levels in patients with hyperthyroidism, it is suggested that the increase in FT4 may be more closely related to the changes in blood lipids than the effect of TSH on the blood lipids.

With the decline in thyroid function, the level of lipids increases gradually (18, 19). In our study, the levels of TC and TG increased significantly. In univariate regression analysis, the risk of dyslipidemia in all subgroups of hypothyroidism increased significantly, but after controlling for confounding factors, only the risk of hyperTG increased by 50.2%.This may be because the age, sex ratio and other confounding factors of patients with hypothyroidism are more different from those of the control group. Regarding HDL, the results have been debated. Although our study found an increased prevalence of low HDL-C in patients with hypothyroidism, the statistical significance disappeared after correcting the confounding factors. The results of Kaliaperumal R et al. showed that the level of HDL-C in patients with hypothyroidism was significantly lower (20). Chen Y et al. analyzed 197 newly diagnosed patients with hypothyroidism and found no significant change in HDL-C (3). These inconsistencies may be due to the different thyroid reference values and severity of hypothyroidism. This study further indicates that patients with hypothyroidism are more likely to have changes in blood lipid profiles of cardiovascular diseases.

Some studies have reported the relationship between subclinical hypothyroidism and blood lipids, but the results are inconsistent (21–23). In our study, TG levels were significantly increased and HDL-C levels were significantly decreased in patients with subclinical hypothyroidism. After full adjustment, we demonstrated significantly increased risks of dyslipidemia, indicating harmful effects of high TSH against dyslipidemia. For instance, the risk of hyperTG was increased by 24.2%, and the risk of low HDL-C was increased by 32.9%, which was similar to the findings previously. Conclusions on subclinical hypothyroidism and HDL-C are different. Regmi et al. reported that TSH levels were not significantly correlated with HDL-C in a subclinical hypothyroidism population (24). McGowan A et al. found that the HDL-C was only decreased in patients with subclinical hypothyroidism and remained normal in patients with hypothyroidism, which was similar to our research conclusion (25). The researchers reported that the activity of cholesterol ester transfer protein (CETP) is decreased in hypothyroid patients, resulting in the accumulation of HDL-C, which is not observed in subclinical hypothyroidism patients. However, subclinical hypothyroidism has a greater effect on blood lipids than subclinical hyperthyroidism, suggesting that the increase in TSH has a stronger correlation with blood lipids. Based on these results, patients with thyroid abnormalities should also pay dynamic attention to changes in blood lipids.

Menopause always leads to changes in hormone status, metabolism and blood lipids. Interestingly, when we performed logistic regression separately in the premenopausal and postmenopausal groups, we clearly observed that the associations between thyroid parameters and lipids were not identical: a significant positive correlation of dyslipidemia with hypothyroidism and subclinical hypothyroidism was only detected in premenopausal females but not in postmenopausal females. Premenopausal females with subclinical hypothyroidism had a significantly higher risk of hyperTG and low HDL-C, but after menopause, females did not demonstrate such significance. Regarding hyperthyroidism, premenopausal females had a significantly decreased risk of hyperTC and hyperLDL-C, and postmenopausal females had a significantly increased risk of low HDL-C. The change in this relationship is associated with the decrease in estrogen secretion and the sharp increase in FSH levels after menopause. Decreased estrogen secretion can lead to a deterioration in blood lipids (26). Recent studies have shown that FSH is the main regulator of fat and energy homeostasis, and an increase in FSH levels is associated with fat accumulation and redistribution and increased cholesterol production (27).Consequently, menopause makes the effect of thyroid function disorder on blood lipids no longer obvious. Therefore, hypothyroidism and subclinical hypothyroidism have a greater impact on blood lipids in premenopausal women. Hyperthyroidism changes from a protective effect on blood lipids before menopause to a harmful effect on blood lipids after menopause. These observations strongly indicate that menopause might interfere with the effect of TH on the lipid profile, which was the most important finding in our study. Based on these results, thyroid screening and modification should be strongly considered in postmenopausal females with hyperthyroidism and premenopausal females with hypothyroidism and subclinical hypothyroidism, but the specific mechanism of the interaction between thyroid disease and menopause still needs to be further studied.

The advantage of this study is that the general population surveyed in the community, which is more representative, the sample size is relatively large, the quality control in the process of data collection is strict, and the potential risk factors are corrected in the multifactor model, which makes the results of our analysis more reliable. The limitation of this study is that its cross-sectional design limits the conclusion of causality; there is a lack of information about the use of lipid-regulating drugs, and consequently, people who are known to have dyslipidemia are excluded.

In summary, this study found hypothyroidism and subclinical hypothyroidism have more significant effects on blood lipids in premenopausal females. Hyperthyroidism is a protective factor against hyperTC and hyperLDL-C in premenopausal females and a risk factor for low HDL-C in postmenopausal females. The effect of subclinical hyperthyroidism on blood lipids was not statistically significant. In sum, this study provides new results for the relationship between thyroid dysfunction and dyslipidemia. The coexistence and interaction of thyroid dysfunction and dyslipidemia need to be confirmed by prospective studies.

## Data Availability Statement

The raw data supporting the conclusions of this article will be made available by the authors, without undue reservation.

## Ethics Statement

The studies involving human participants were reviewed and approved by the Ethics Committee of China Medical University. The patients/participants provided their written informed consent to participate in this study.

## Author Contributions

YH, CW, LZ, JZ, and MZ contributed equally to this work. YH and CW performed the data analyses and drafted the manuscript. LZ, JZ, MZ, YL, DT, and the Thyroid Disorders, Iodine Status and Diabetes Epidemiological Survey Group participated in the epidemiological investigations. WT and ZS conceived and designed the study and interpreted the results. All authors contributed to the article and approved the submitted version.

## Funding

This work was supported by the Research Fund for Public Welfare from National Health and Family Planning Commission of China (Grant No. 201402005) and the Clinical Research Fund of Chinese Medical Association (Grant No. 15010010589).

## Conflict of Interest

The authors declare that the research was conducted in the absence of any commercial or financial relationships that could be construed as a potential conflict of interest.

## Publisher’s Note

All claims expressed in this article are solely those of the authors and do not necessarily represent those of their affiliated organizations, or those of the publisher, the editors and the reviewers. Any product that may be evaluated in this article, or claim that may be made by its manufacturer, is not guaranteed or endorsed by the publisher.
